# Compliance With Multidisciplinary Care Standards in a Psychiatric Inpatient Unit: A Clinical Audit From a Tertiary Hospital in the United Arab Emirates

**DOI:** 10.1192/bjo.2026.11689

**Published:** 2026-06-30

**Authors:** Mohammed Al Ahbabi, Khaled Jawabri, Alyazia Al Kaabi, Fadwa Al Mugaddam, Syed Fahad Javaid

**Affiliations:** 1 Behavioural Sciences Institute, Al Ain, UAE; 2 Department of Psychiatry, College of Medicine and Health Sciences, United Arab Emirates University, Al Ain, UAE

## Abstract

**Aims:**

Multidisciplinary care is essential for safe and effective inpatient psychiatric treatment. Involvement of social work and psychology services supports psychosocial assessment, risk management, discharge planning, and continuity of care. This audit assessed compliance with local guidelines for multidisciplinary inpatient care, informed by the Royal College of Psychiatrists’ Core Standards for Inpatient Mental Health Services (Centre for Quality Improvement), which emphasise timely multidisciplinary involvement, including psychology and social work input, and clear documentation in multidisciplinary team reviews. We aimed to identify good practices and any gaps in early referral and documentation.

**Methods:**

A retrospective audit at the Behavioural Sciences Institute (BSI), Al Ain Hospital, United Arab Emirates, reviewed electronic records of 193 psychiatric admissions. Four standards from the Royal College of Psychiatrists’ Core Standards were assessed: (1) social work referral within a week; (2) psychology referral within a week of admission; (3)documentation of social work input in multidisciplinary team (MDT notes; and (4) psychology input documented in MDT notes. Compliance was measured by percentage, stratified by unit type (male, female, forensic) to explore practice variation. Descriptive data analysis was used.

**Results:**

Social work referrals within one week were completed in 29.0% of admissions, and psychology referrals in 32.6%. Documentation rates were higher but still suboptimal: social work input was recorded in 38.3% of cases, and psychology in 44.0%. There was variation between units. The male unit had the lowest early referral compliance, with social work and psychology orders in 16.0% and 22.0% of cases, respectively. MDT documentation was higher but incomplete. The female unit performed better, with social work orders in 47.5% and psychology orders in 50.8%, though psychology documentation was low at 32.8%. In the forensic unit, early referrals were moderate for social work (34.4%) and low for psychology (25.0%), but MDT documentation was the highest, with social work noted in 75.0% and psychology in 56.3%. Overall, MDT documentation rates exceeded early referral rates, indicating delayed multidisciplinary involvement.

**Conclusion:**

This audit found gaps in referral practices for social work and psychology in psychiatric inpatient care, especially early referral within a week of admission. Documentation in MDT notes was more frequent but still below standards, indicating delayed engagement. To improve care, structured admission pathways, automated referrals, standardised documentation, and staff education are needed.

No financial sponsorship was received for this project.

